# The Current State of Evidence Regarding Audiologist-Provided Cognitive Behavioural Therapy for the Management of Tinnitus: A Scoping Review

**DOI:** 10.3390/audiolres14030035

**Published:** 2024-04-30

**Authors:** Louise A. Burke, Amr El Refaie

**Affiliations:** Audiology, School of Clinical Therapies, College of Medicine and Health, University College Cork, T12 EK59 Cork, Ireland

**Keywords:** tinnitus, cognitive behavioural therapy, CBT, audiologist, scoping review

## Abstract

Background: Cognitive behavioural therapy (CBT) for tinnitus management is effective and widely recommended by national and international practice guidelines. However, all the evidence for CBT so far has come from Psychologist-led programs, and the potential role of Audiologists in providing CBT for tinnitus remains an important consideration. Objectives: This study sets out to systematically map the body of literature relating to Audiologist-provided CBT for tinnitus, in order to summarise the current state of evidence and determine directions for future research. Eligibility criteria: Sources were eligible for inclusion if they addressed the concept of Audiologist-provided CBT. No restrictions were imposed on the date of publication. Only sources published in English were included. Sources of evidence: A wide range of primary and secondary literature sources were sought. Charting methods: Data from included sources were charted systematically using a pre-designed data charting form. Results: Of the 267 identified sources, 30 were included in this review. This included both primary and secondary literature sources. Primary sources were compared and showed variation across Audiologist-provided CBT programs both in terms of procedural details and from a research standpoint. Conclusions: A growing body of evidence has addressed the concept of Audiologist-provided CBT. Directions for future research include further primary research with an increased focus on face-to-face Audiologist-provided CBT, and a comparison of the outcomes of Audiologist-provided vs. Psychologist-provided CBT.

## 1. Introduction

Tinnitus has been defined as the ‘percept of a sound or sounds in the ear or head without an external source’, while bothersome tinnitus has been further described as a ‘negative emotional and auditory experience, associated with, or described in terms of, actual or potential physical or psychological harm’ [1]. Tinnitus is estimated to affect 5–43% of adults worldwide [2], with 1 in 15 of those describing it as bothersome [3]. Bothersome tinnitus is associated with adverse effects like sleep disturbances [4], impaired cognitive functioning [5], and anxiety and depression [6,7].

Even prior to the publication of McKenna and colleagues’ [8] cognitive-behavioural model of tinnitus, cognitive behavioural therapy (CBT) had found application in tinnitus management [9]. The aim of CBT for tinnitus is to target the tinnitus reaction as opposed to the perception. Both ‘traditional’ second-wave CBT programs, which seek to change the recipient’s thoughts, emotions, and behaviours around tinnitus, and newer third-wave CBT programs (e.g., acceptance and commitment therapy, mindfulness-based cognitive therapy), which emphasise acknowledgement and acceptance of tinnitus, are in use [9]. There is strong evidence that CBT can improve tinnitus-related quality of life and reduce distress and annoyance due to tinnitus [9,10,11]. Therefore, it is widely recommended in the management of tinnitus [1,12,13].

Despite this, studies such as those of McFerran et al. [14], Hoare et al. [15], and Gander et al. [16] have highlighted barriers to tinnitus patients accessing psychological management, including CBT, in the UK. These include a shortage of mental health professionals and ineffective referral pathways to Psychology. While data from other countries are lacking, similar barriers may exist globally. One possible solution is for appropriately trained Audiologists to deliver CBT, a model of care which has been suggested in the UK [17]. Audiology clinics are often the first point of referral for patients who present to their GP with tinnitus [14,18]. Additionally, Audiologists possess specialist knowledge of the auditory system and relative understanding of the generation of tinnitus, which may place them as the most appropriate professionals to deliver any kind of tinnitus intervention, psychological or otherwise.

However, the efficacy of CBT when provided by Audiologists is unknown [19], and all of the current evidence for CBT in tinnitus management comes from Psychologist-led programs. Recognising this, the National Institute of Clinical Excellence [13] recommends CBT as an intervention for tinnitus in the UK but stipulates that it must be delivered by a psychologist, although neither the European guidelines [1] nor the British Society of Audiology (BSA) guidelines [12] specify who should deliver the intervention. In 2013, Baguley, McFerran, and Hall [20] identified the efficacy of Audiologist-provided CBT and psychological therapies more generally as one of the top 10 uncertainties relating to tinnitus assessment, diagnosis, and treatment. In the same year, The James Lind Alliance Tinnitus Priority Setting Partnership [21] listed the efficacy of Audiologist-delivered CBT as one of the top 10 clinical research priorities, calling for empirical research on this topic.

A decade after this prioritisation, there remains no review or summary of the literature in this area. This review sought to address the research question ‘what is the current state of evidence regarding Audiologist-provided CBT for the management of tinnitus?’ According to Munn et al. [22], where a body of literature has not previously been reviewed and the research question of interest has a relatively broad focus, a scoping review is a useful methodology. Therefore, a scoping review was undertaken, with the following objectives:

1. To identify all sources of evidence relating to Audiologist-provided CBT, in order to determine the volume, range, and type of evidence available, identify any gaps in the current literature, and provide recommendations for future research;
2. To map details relating to (a) the content and structure of Audiologist-provided CBT programs (procedural details), and (b) how research has been conducted on this topic (study details), in order to inform future research.

## 2. Materials and Methods

This review was conducted as per the Joanna Briggs Institute (JBI) methodological framework for scoping reviews [23,24], and reported as per the PRISMA-Scr guidelines [25].

### 2.1. Protocol and Registration

No a priori protocol was published for this review because it was completed in partial fulfilment of a postgraduate degree and was therefore an iterative, learning process which required some flexibility of conduct.

### 2.2. Eligibility Criteria

#### 2.2.1. Population, Concept, and Context

In order to be eligible for inclusion in this review, sources of evidence must have addressed the concept of Audiologist-provided CBT for tinnitus. Sources were excluded which deviated from any aspect of this concept (i.e., the intervention was provided by any professional other than an Audiologist, the intervention was delivered for the management of any condition other than tinnitus, or the intervention description was inconsistent with CBT). Sources describing either second- or third-wave CBT were included, but those describing cognitive or behavioural interventions alone, or other non-CBT-based interventions for tinnitus, were excluded.

There were no restrictions imposed regarding contextual factors such as the setting of the intervention or mode of delivery. However, sources describing internet-based CBT (iCBT) were only included provided that an Audiologist guided the intervention; those describing completely unguided iCBT were excluded. Regarding both iCBT and face-to-face CBT, sources were included if an Audiologist was the primary professional involved in the delivering intervention, even if it was primarily self-directed by participants.

#### 2.2.2. Types of Sources

Eligible for inclusion were analytical cross-sectional studies, online books and book chapters, case control studies, case reports, case series, cohort studies, literature reviews, qualitative research, quasi-experimental studies, randomised controlled trials (RCTs), scoping reviews, systematic reviews, protocols, text and opinion pieces, and grey literature (i.e., unpublished sources, pre-print articles, theses and dissertations, clinical trial protocols, conference abstracts describing unpublished results, and clinical trial registrations for unpublished trials).

#### 2.2.3. Other

Only studies published in English were included due to time constraints associated with this review. No limitations were placed on the date of publication.

### 2.3. Information Sources

The following databases were searched for relevant articles: CINAHL plus (via EBSCOhost), Cochrane Library, Embase, Medline (via EBSCOhost), APA PsycINFO (via EBSCOhost), PubMed, ScienceDirect, Scopus, Web of Science Core Collection, CORE, ResearchGate, ClinicalTrials.gov, World Health Organisation International Clinical Trials Registry Platform, and EU Clinical Trials Register. All were searched from inception to present.

### 2.4. Search

An initial limited search of two databases, CINAHL plus and Medline, was conducted in October 2022. The key words, index terms, and text words used in the titles and abstracts of the relevant articles returned were assessed and used to develop a search string (Appendix A) which was adapted for use across all databases. The search string as used in one database is provided in Appendix B as an example.

The full search was conducted on 19 January 2023. No language restrictions were imposed on the search. Instead, papers were assessed for eligibility based on the language of publication at the study selection stage, as recommended in order to promote transparency [26]. Where trial registrations were found and the trial was complete, a separate search was conducted to locate the publication relating to the trial, and, where found, this was included instead of the trial registration.

The reference lists of all included papers were searched for additional relevant papers which were not identified through the aforementioned search strategies. Where required, authors were contacted to obtain full-text articles.

### 2.5. Selection of Sources of Evidence

All returned articles from CINAHL plus, Embase, Medline, PsycINFO, PubMed, ScienceDirect, Scopus, and Web of Science Core Collection were downloaded into EndNote online. Results from all other platforms were stored in Microsoft excel. After removing duplicates, all remaining articles were screened by title and abstract. Those which met the inclusion criteria were read in full text, and those which still met the inclusion criteria by full text were included in the review. The selection process was conducted independently by LB.

### 2.6. Data Charting

A data extraction form, based on a template published by the JBI [27], was developed by LB and reviewed by AER. Having reached agreement on the included categories, the form was piloted on three papers and slight modifications were made. The finalised form is shown in Appendix C. Data charting was conducted independently by LB.

### 2.7. Data Items

Individual data items obtained from each source were source type, publication status, year of publication, aim(s) of the source as they relate to the review topic, and, as appropriate, study design, country of conduct, procedural details (i.e., content of the CBT intervention, details of provider, training received to provide CBT, intervention setting, unit of delivery, mode of delivery, role of the Audiologist, frequency of CBT sessions, duration of individual CBT sessions, and duration of the CBT program), and study details (i.e., eligibility criteria, sample size, mean age and gender distribution of the sample, and information about tinnitus duration and severity among the sample).

### 2.8. Synthesis of Results

The total number of identified sources, number in each category of evidence type, mode year of publication and range, and number of sources published and unpublished were reported. The subset of empirical research articles was further categorised by study design used and the number of studies in each category was reported. In the subset of intervention studies, data relating to procedural details of the intervention and study details were summarised across studies, and similarities and contrasts were highlighted.

## 3. Results

### 3.1. Selection of Sources of Evidence

**Figure 1:**
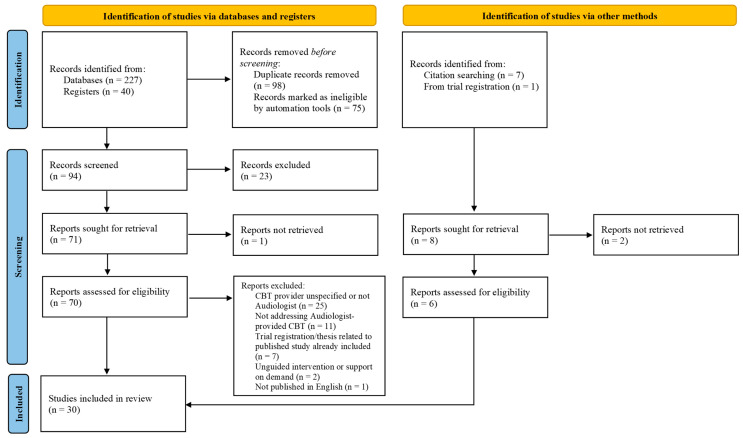
Flow diagram of search process.

The study selection process is depicted in a PRISMA flow diagram (Figure 1) [28]. A total of 267 sources of evidence were identified in the initial search. After removing duplicates, 94 sources remained and were screened by title and abstract. Of those, 70 sources were retrieved and assessed for eligibility by full text, and 24 sources met the inclusion criteria. An additional eight sources were identified by searching the reference lists of included studies and searching for published trials based on trial registrations. Six of those were retrieved in full text and met the inclusion criteria. Therefore, a total of 30 sources of evidence were included in this review.

### 3.2. Characteristics of Sources of Evidence

The 30 sources comprised 17 empirical research articles, four text and opinion pieces, two book chapters, two descriptive articles, two clinical trial protocols, one book, one literature review, and one clinical trial registration (Table 1). Among the 17 empirical research articles, a variety of different study designs and methodologies were implemented. At the time of writing, and excluding the clinical trial registration, 27 sources were published and two were unpublished. The year of publication ranged across sources from 1986 to 2021, with the mode year being 2018 (seven sources).

### 3.3. Results of Individual Sources of Evidence

Sixteen intervention studies assessed Audiologist-provided CBT programs. In terms of procedural details, these studies can be categorised into four groups (i.e., one group of studies relating to a face-to-face CBT intervention led by Aazh [29,30,32], one group of studies relating to an iCBT intervention led by Beukes [34,35,36,37,38,39,40,41,43,44,45], and two individual studies conducted by Taylor et al. [54] and Tay [52]) (detailed in Table 2 and Table 3).

### 3.4. Synthesis of Results

#### 3.4.1. Key Characteristics

##### Setting, Unit, and Mode of Delivery

Most studies used an internet-based intervention which was delivered via weekly online modules, including all of the iCBT interventions led by Beukes [34,35,36,37,38,39,40,41,43,44,45], and Tay’s [52] iCBT intervention. Fewer programs employed face-to-face delivery, those being the Aazh-led CBT interventions [29,30,32] and Taylor et al.’s [54] intervention. Face-to-face CBT programs were all conducted within hospital-based Audiology departments. All studies used either a participant-led or one-to-one approach, with no report of group CBT sessions.

##### Dosage

The frequency and duration of CBT sessions, and duration of the program, varied across studies. For example, the interventions described by the Aazh-led group of studies [29,30,32] involved six hour-long sessions across 2–4 months, and in the Beukes-led iCBT interventions [34,35,36,37,38,39,40,41,43,44,45], 2–3 modules were released per week for 8 weeks. In comparison, Taylor et al. [54] provided 1–3 sessions but did not specify the length of each session or timeframe across which they were provided, while Tay [52] provided no information regarding dosage.

##### Training and Role of Audiologists

Training of Audiologists varied between studies and tended to match the level of involvement they had in the intervention. Audiologists in the Aazh-led group of studies [29,30,32] undertook thorough multi-component training across 150 h, followed by six months of clinical supervision. Components included training around client-centred counselling, motivational interviewing, and CBT. The CBT program in these studies was fully delivered by Audiologists in a face-to-face format.

Audiologists in Taylor et al. [54] attended a two-day training workshop and were also expected to fully deliver face-to-face CBT intervention, but their program was comparatively less involved, and participants underwent fewer CBT sessions. Finally, Audiologists in the group of iCBT studies led by Beukes [34,35,36,37,38,39,40,41,43,44,45], and those in Tay’s study [52], received no formal CBT training. Their role was to guide participants through the CBT program, but it was ultimately participant-led.

##### Intervention Content

A wide range of CBT components were used, borrowed from both second-wave (e.g., thought analysis, cognitive restructuring) and third-wave (e.g., focusing techniques, attention, monitoring, and acceptance) CBT approaches. Furthermore, some components were more cognitively rooted (e.g., identifying negative thinking patterns, diary of thoughts and feelings), while others primarily targeted behaviours (e.g., behavioural exposure techniques, managing fear and avoidance behaviours). Only the group of iCBT interventions led by Beukes [34,35,36,37,38,39,40,41,43,44,45] specified the inclusion of both recommended and optional techniques, the latter of which mostly related to sound enrichment and sleep hygiene. 

**Table 2:** Procedural Details of Audiologist-Provided CBT Programs.

**Group of CBT studies led by Aazh:** Aazh et al. [29]; Aazh and Moore [30]; Aazh, Bryant, and Moore [32]				
**CBT Techniques Included**	**Training Received to Provide CBT**	**Details of Delivery**	**Dosage**	**Outcomes Assessed (Measure Used)**
Socratic questioning, guided discovery, behavioural experiments, education, and filling in diaries of thoughts and feelings.	Attendance at a tinnitus master class which included elements of CBT, counselling, and motivational interviewing training. This involved 30 h of direct contract, 120 h of independent learning, 6 months of observation and supervised clinical practice, and ongoing coaching, support, clinical supervision, and informal training.	Setting: Audiology department of one NHS hospital Unit: Individual sessions Mode: Face-to-face Role of audiologists: To deliver all parts of the intervention	Six 1-h CBT sessions were provided across a 2–4-month time period, with typically one session per week.	Aazh et al. [28]; Aazh and Moore [29] Tinnitus severity (THI), hyperacusis severity (HQ), depression (HADS), tinnitus qualities (VAS loudness, annoyance, and effect on life), insomnia (ISI)
				Aazh, Bryant, and Moore [31] Tinnitus severity (THI), hyperacusis severity (HQ), general anxiety (GAD-7), depression (PHQ-9), tinnitus qualities (VAS loudness, annoyance, and effect on life), insomnia (ISI)
**Group of CBT studies led by Beukes:** *UK-based studies:* Beukes et al. [34]; Beukes, Baguley, et al. [35]; Beukes, Allen, et al. [36]; Beukes, Manchaiah, Baguley, et al. [37]; Beukes, Manchaiah, Davies, et al. [38]; Beukes, Andersson, et al. [39] *USA-based studies:* Beukes et al. [40]; Beukes et al. [41]; Beukes, Andersson, and Manchaiah [43]; Beukes, Andersson, Fagelson, and Manchaiah [44]; Beukes, Andersson, and Manchaiah [45] **Related protocol:** Beukes et al. [33]				
**CBT Techniques Included**	**Training Received to Provide CBT**	**Details of Delivery**	**Dosage**	**Outcomes Assessed (Measure Used)**
Recommended modules: Tinnitus overview, deep relaxation, positive imagery, diaphragmatic breathing, reinterpreting tinnitus, focusing techniques, rapid relaxation, thought analysis, relaxation in daily routines, relaxation in stressful situations, cognitive restructuring, exposure to tinnitus, reviewing helpful techniques, and maintenance and relapse preventio (additional mindfulness module in USA-based studies only). Optional modules: Sound enrichment, sleep guidelines, concentration tips, reducing sound sensitivity, and hearing tactics.	No formal CBT training. Supervision was provided by a clinical psychologist who was experienced in tinnitus management.	Setting: Online Unit: Individual Mode: Internet-based modules Role of audiologist: To guide participants to self-direct the intervention. Audiologists contacted participants, introduced module content, monitored progress, gave feedback and encouragement, and answered questions.	2–3 modules were released weekly over an 8-week period. Each module involved 10–20 min of reading and additional daily practising.	UK-based studies Tinnitus severity (TFI), general anxiety (GAD-7), depression (PHQ-9), insomnia (ISI), hyperacusis severity (HQ), hearing handicap (HHIA-S), quality of life (SWLS), and cognitive function (CFQ) USA-based studies Tinnitus severity (TFI), general anxiety (GAD-7), depression (PHQ-9), insomnia (ISI), cognitions related to tinnitus (TCQ), HRQoL (EQ-5D-5L), and tinnitus, hearing, and hyperacusis-related problems (THS)
**Individual study:** Tay [52]				
**CBT Techniques Included**	**Training Received to Provide CBT**	**Details of Delivery**	**Dosage**	**Outcomes Assessed (Measure Used)**
Behavioural exposure techniques, mindfulness-based stress reduction, relaxation exercises, cognitive restructuring, sound enrichment, refocusing, concentration tips, and identifying negative thinking patterns.	No specific CBT training was reported.	Setting: Online Unit: Individual Mode: Internet or app-based modules Role of audiologist: To guide participants to self-direct the intervention and to assist in some CBT exercises e.g., facilitating the identification of negative thoughts	Not reported	Tinnitus severity (TFI, THI, and TRQ)
**Individual study:** Taylor et al. [54] **Related protocol:** Taylor et al. [53]				
**CBT Techniques Included**	**Training Received to Provide CBT**	**Details of Delivery**	**Dosage**	**Outcomes Assessed (Measure Used)**
Goal-setting, rapid relaxation, managing fear and avoidance behaviours, changing unhelpful negative thoughts and beliefs, promotion of physical exercise, promotion of good sleep habits, sound enrichment, and attention, monitoring, and acceptance.	Attendance at a 2-day workshop and use of a specially designed manual.	Setting: Audiology department of three NHS hospitals Unit: Individual Mode: Face-to-face Role of audiologist: To deliver all parts of the intervention	1–3 CBT sessions (mean = 2.75) were provided. The frequency and length of the sessions were not reported.	Tinnitus severity (TFI), cognitions related to tinnitus (TCQ), and psychological well being and distress (CORE-OM).

Note. All measures listed were assessed pre- and post-intervention. Abbreviations and acronyms used: CBT = cognitive behavioural therapy; CFQ = Cognitive Failures Questionnaire [59]; CORE-OM = Clinical Outcomes Routine Evaluation-Outcome Measure [60]; EQ-5D-5L = European Quality of Life 5 Dimensions 5 Level Version [61]; GAD-7 = Generalized Anxiety Disorder [62]; HADS = Hospital Anxiety and Depression Scale [63]; HHIA-S = Hearing Handicap Inventory for Adults-Screening version [64]; HRQoL = health-related quality of life; HQ = Hyperacusis Questionnaire [65]; ISI = Insomnia Severity Scale [66]; NHS = National Health Service; PHQ-9 = Patient Health Questionnaire [67]; SWLS = Satisfaction With Life Scale [68]; TCQ = Tinnitus Cognitions Questionnaire [69]; TFI = Tinnitus Functional Index [70]; THI = Tinnitus Handicap Inventory [71]; THS = Tinnitus and Hearing Survey [72]; TRQ = Tinnitus Reaction Questionnaire [73]; VAS = visual analogue scale; VAS of tinnitus loudness, annoyance, and effect on life [74].

**Table 3:** Eligibility Criteria and Participants Characteristics Across Studies.

Author (Year)	Eligibility Criteria	Participant Characteristics	
		**Sample size and demographic information**	**Tinnitus severity and duration at baseline**
Aazh et al. (2016) [29]	Not applicable	**Sample size:** *N* = 92 **Age:** *M* = 62 years (*SD* = 15) **Gender:** 63%m, 37%f	**THI:** *M* = 47 (*SD* = 24) **Duration:** *M* = 10 years (*SD* = 10)
Aazh and Moore (2018) [30]	Not applicable	**Sample size:** *N* = 68 **Age:** *M* = 53 years (*SD* = 13) **Gender:** 57%f, 43%m	**THI:** M = 61 (*SD* = 18) **Duration:** Not reported
Aazh, Bryant, and Moore (2019) [32]	Not applicable	**Sample size:** *N* = 40 **Age:** *M* = 48 years (*SD* = 14) **Gender:** 55%f, 45%m	**THI:** *M* = 62 (*SD* = 16) **Duration:** Not reported
Beukes et al. (2017) [34]	Inclusion criteria: Aged ≥ 18 years; living in the UK; ability to read and type in English; tinnitus duration of ≥3 months. Exclusion criteria: Major self-reported medical or psychiatric disorder; uninvestigated tinnitus of a pulsatile, objective, or unilateral nature; tinnitus resulting from a medical disorder; undergoing any tinnitus therapy.	**Sample size:** *N* = 37 **Age:** *M* age range = 50-59 years (*SD* = 1) **Gender:** 51%f, 49%m	**TFI:** *M* = 56 (*SD* = 18) **THI-S:** *M* = 23 (*SD* = 8) **Duration:** Mo = 1-5 years
Beukes, Baguley, et al. (2018) [35]; Beukes, Manchaiah, Baguley, et al. (2018) [37]	Inclusion criteria: Aged ≥ 18 years; living in the UK; ability to read and type in English; tinnitus duration of at least 3 months; TFI score of ≥25. Exclusion criteria: Major self-reported medical, psychiatric, or mental disorder; uninvestigated tinnitus of a pulsatile, objective, or unilateral nature; tinnitus resulting from a medical disorder still under investigation; undergoing any tinnitus therapy.	**Sample size:** *N* = 146 **Age:** *M* = 56 years (SD = 13) **Gender:** 57%m, 43%f	**TFI:** *M* = 60 (*SD* = 18) **Duration:** *M* = 12 years (*SD* = 12)
Beukes, Allen, et al. (2018) [36]	Inclusion criterion: Completed iCBT intervention reported in Beukes, Baguley, et al. (2018).	**Sample size:** *N* = 139 **Age:** *M* = 58 years (*SD* = 13) **Gender:** 56%m, 44%f	**TFI:** *M* = 59 (*SD* = 17) **Duration:** *M* = 12 years (*SD* = 11)
Beukes, Manchaiah, Davies, et al. (2018) [38]	Inclusion criterion: Completed iCBT intervention reported in Beukes, Baguley, et al. (2018).	**Sample size:** *N* = 15 **Age:** *M* = 59 years (*SD* = 8) **Gender:** 53%f, 47%m	**TFI:** M = 58 (*SD* = 16) **Duration:** *M* = 9 years (*SD* = 9)
Beukes, Andersson, et al. (2018) [39]	Inclusion criteria: Aged ≥ 18 years; regular access to computer and internet. Exclusion criteria: Any major self-reported medical or psychiatric conditions; undergoing any tinnitus therapy.	iCBT group **Sample size:** *n* = 46 **Age:** M = 51 years (*SD* = 12) **Gender:** 63%m, 37%f Face-to-face group **Sample size:** *n* = 46 **Age:** *M* = 55 years (*SD* = 12) **Gender:** 57%m, 43%f	iCBT group **TFI:** *M* = 55 (*SD* = 22) **THI:** *M* = 45 (*SD* = 23) **Duration:** *M* = 5 years (*SD* = 9) Face-to-face group **TFI:** *M* = 57 (*SD* = 21) **THI:** *M* = 47 (*SD* = 20) **Duration:** *M* = 8 years (*SD* = 10)
Beukes et al. (2021a) [40]	Inclusion criteria: Aged ≥ 18 years; living in Texas, USA; ability to read and type in English or Spanish; tinnitus duration of at ≥3 months; TFI score of ≥25. Exclusion criteria: PHQ-9 scores of ≥15; major self-reported medical or psychiatric disorder; tinnitus of a pulsatile, objective, or unilateral nature which is uninvestigated or currently under investigation; undergoing any tinnitus therapy.	**Sample size:** *N* = 27 **Age:** *M* = 56 years (*SD* = 10) **Gender:** 67%f, 33%m	**TFI:** *M* = 58 years (*SD* = 15) **Duration:** *M* = 12 years (*SD* = 13)
Beukes et al. (2021b) [41]	Inclusion criteria: Aged ≥ 18 years; living in Texas, USA; ability to read and type in English; access to a computer, internet, and email; self-perceived need of tinnitus intervention. Exclusion criteria: Major self-reported medical condition or treatment; tinnitus of a pulsatile, objective, or unilateral nature which is uninvestigated or currently under investigation; undergoing tinnitus therapy.	iCBT group **Sample size:** *n* = 63 **Age:** *M* = 55 years (*SD* = 13) **Gender:** 59%f, 41%m Relaxation group **Sample size:** *n* = 63 **Age:** *M* = 57 years (**SD** = 13) **Gender:** 60%m, 40%f	iCBT group **TFI:** *M* = 50 (*SD* = 27) **Duration:** *M* = 10 years (*SD* = 11) Relaxation group **TFI:** *M* = 49 (*SD* = 26) **Duration:** *M* = 15 years (*SD* = 14)
Beukes, Andersson, Fagelson, and Manchaiah (2022) [44]; Beukes, Andersson, and Manchaiah (2021) [43]	As described in Beukes et al. (2021a).	**Sample size:** *N* = 158 **Age:** *M* = 57 years (*SD* = 12) **Gender:** 51%f, 49%m	**TFI:** Not reported **Duration:** *M* = 14 years (*SD* = 14)
Beukes, Andersson, and Manchaiah (2022) [45]	Inclusion criterion: Completed iCBT intervention reported in Beukes et al. (2021b) or Beukes, Andersson, Fagelson, & Manchaiah (2022).	**Sample size:** *N* = 132 **Age:** *M* = 56 years (*SD* = 13) **Gender:** 56%f, 44%m	**TFI:** *M* = 54 (*SD* = 21) **Duration:** *M* = 12 years (*SD* = 15)
Taylor et al. (2020) [53]	Inclusion criteria: Aged ≥ 18 years; capacity to consent; sufficient mobility to attend clinics; TFI score ≥ 25; willing to share experiences of participating. Exclusion criteria: Tinnitus with a medically treatable origin; unable to communicate in English; participated in other tinnitus management research after consenting to this study.	Manualized psychological care group **Sample size:** *n* = 11 **Age:** *M* = 59 years (*SD* = 11) **Gender:** 70%m, 30%f Treatment as usual group **Sample size:** *n* = 8 **Age:** *M* = 44 years (*SD* = 18) **Gender:** 63%m, 37%f	Manualized psychological care group **TFI:** *M* = 67 (*SD* = 24) **Duration:** Not reported Treatment as usual group **TFI:** *M* = 50 (*SD* = 24) **Duration:** Not reported
Tay (Unpublished work) [51]	Inclusion criteria: Aged ≥ 18 years; living in the Republic of Indonesia; ability to read and type in Bahasa Indonesia or English; referred by appropriately certified healthcare professional; TFI score of ≥50. Exclusion criteria: None reported.	**Sample size:** *N* = 40 **Age:** *M* = 47 years (*SD* = 16) **Gender:** 63%m, 38%f	**TFI:** *M* = 60 (*SD* = 5) **Duration:** *M* = 16 years (*SD* = 13)

Note. All figures reported to 0 decimal places. Abbreviations and acronyms used: iCBT = internet-based cognitive behavioural therapy; *M* = mean; Mo = mode; *N* = size of entire sample; *n* = size of subsample; PHQ-9 = Patient Health Questionnaire [67]; *SD* = standard deviation; TFI = Tinnitus Functional Index [70]; THI = Tinnitus Handicap Inventory [71]; THI-S = Tinnitus Handicap Inventory–screening version [75]; %f—percentage of females; %m = percentage of males in the sample. No further/alternative categorisations of gender were used in any study.

###### Outcome Assessment

In all programs, outcomes were measured at baseline and again immediately post intervention. Tinnitus severity was the primary outcome in all studies and was most often measured using the Tinnitus Functional Index (TFI) [70]. Other frequently used measures were the Tinnitus and Hearing Survey tinnitus subscale [72] and the Tinnitus Cognitions Questionnaire [69]. Associated symptoms frequently assessed were insomnia, anxiety, depression, and hyperacusis.

#### 3.4.2. Study Details

##### CBT Participants

Eligibility criteria employed in Beukes et al. [34], Beukes, Baguley, et al. [35], Beukes, Andersson, et al. [39], Beukes et al. [40,41], Tay [52], and Taylor et al. [54] generally specified that participants be adults with subjective, chronic, bothersome tinnitus, and no major psychiatric comorbidities. As a result, most participants obtained baseline TFI scores consistent with severe functional impact (i.e., ≥50) and the average tinnitus duration across samples ranged from 5 to 15 years. In contrast, participants of the Aazh-led studies [23,29,30] were real patients attending a specialist tinnitus and hyperacusis clinic. Therefore, strict eligibility criteria were not applied, but patients were pre-screened by interview and if it was felt that their tinnitus and/or hyperacusis did not interfere with their daily activities or mood, they were discharged. Average baseline scores on the Tinnitus Handicap Inventory [71] in these studies corresponded to a moderate–severe handicap.

##### Intervention Comparators

Audiologist-provided CBT was most frequently compared to passive intervention or no intervention, either by comparing pre- and post-treatment scores in the same group (e.g., [30,35,36,40,52]) or by utilising a weekly monitoring delayed intervention group [34,43]. Only three studies compared Audiologist-provided CBT to other active interventions: Beukes et al. [41] compared Audiologist-guided iCBT to Audiologist-guided internet-based applied relaxation; Beukes, Andersson, et al. [39] compared Audiologist-guided iCBT to Audiologist-delivered face-to-face treatment as usual; and Taylor et al. [53] compared Audiologist-delivered face-to-face CBT to face-to-face treatment as usual.

## 4. Discussion

### 4.1. Summary of Evidence

The aim of this scoping review was to map the current body of literature relating to Audiologist-provided CBT, with a particular focus on (a) the extent, volume, range, and type of evidence available, and (b) procedural and study-related details of such programs, in order to establish the current state of the literature and guide future research.

In total, 30 sources of evidence were identified which addressed the concept of Audiologist-provided CBT, published across a 35-year timespan. The earlier work is generally more theoretical in nature, while the more recent publications are increasingly empirical in their focus. This may be in response to calls for empirical research on this topic [20,21]. It seems there is growing interest in the topic given the increasing number of publications in the past decade compared to previously, although note that almost half of those were published by Beukes and her research group. As a result, almost all of the empirical research on this topic was conducted in the UK or the USA, leaving a significant research gap on a global scale.

Procedural details of Audiologist-provided CBT programs were relatively homogenous within research groups (e.g., Aazh and colleagues [29,30,32]; Beukes and colleagues [34,35,36,37,38,39,40,41,43,44,45]) but variable across groups in terms of setting, unit, and mode of delivery, training and role of the Audiologist, dosage, CBT content, and outcomes assessed. One trend which did emerge is a predominance of Audiologist-provided iCBT programs across the literature, which were guided by Audiologists but ultimately participant-led, and comparatively few face-to-face programs were described.

There is a considerable body of research available which has investigated the efficacy, acceptability, and/or feasibility of Audiologist-provided CBT. These studies primarily used pre-test post-test designs to assess tinnitus outcomes in adults with severe, chronic tinnitus. Notably, most of these studies excluded individuals with significant mental health problems. This may represent a viable and appropriate model of Audiologist-provided care for tinnitus, as one of the main arguments against Audiologist-provided CBT is that Audiologists are not equipped to deal with significant comorbid mental health issues [48]. Indeed, Audiologists who have been involved in delivering CBT to tinnitus patients have reported similar concerns [54]. This presents important considerations for future research in terms of screening for clinical anxiety and depression, and having a Psychologist available to supervise clinical practice and take over a patient’s care where necessary.

### 4.2. Directions for Future Research

The current body of literature regarding Audiologist-provided CBT is an expanding and encouraging collection, but important limitations and knowledge gaps are evident. In terms of primary research, perhaps the most important research question to address is the efficacy of Audiologist-provided CBT compared to Psychologist-provided CBT, given that Psychologists are both the usual [19] and recommended [13] providers of CBT for tinnitus management. Comparisons of Audiologist-provided CBT to other active Audiologist-provided interventions are also lacking and would provide valuable information about the relative efficacy of such interventions, while controlling for placebo effects. Based on the search of clinical trial registrations undertaken as part of this review, there are currently no planned trials which would achieve either of these aims.

If Audiologist-provided CBT is found to be at least as effective as its alternatives, then evidence would be needed regarding the relative efficacy of specific aspects of the intervention. For example, comparisons of face-to-face CBT versus iCBT, and of individual versus group delivery, would be valuable in informing resource decisions. More information about the most effective components of Audiologist-provided CBT interventions would also be useful for designing future programs. Furthermore, assessing the long-term stability of any positive intervention effects observed is an important consideration. If unequivocal evidence can be gathered for the efficacy of Audiologist-provided CBT for adults with bothersome tinnitus but no significant mental health issues, then research with currently underrepresented groups, such as individuals with co-existing mental health problems and paediatric samples, may be appropriate.

Currently, a narrative systematic review is feasible and would represent a valuable contribution to the literature. Specifically, the question of the efficacy of Audiologist-provided CBT in improving the tinnitus reaction and associated outcomes in adults with chronic, severe tinnitus and no mental health comorbidities could be addressed. The feasibility and acceptability of such programs are also important considerations which could be explored in a systematic review.

While a meta-analysis may not currently be feasible, the research on this topic is relatively convergent regarding the outcomes being assessed and the measures used, with the TFI and the Insomnia Severity Index being most widely used. Therefore, for consistency and to enhance comparability, future research may consider using these questionnaires, and indeed both are recommended for clinical practice by the BSA [12]. They should be integrated with Hall and colleagues’ [76] core outcome set for assessing the effects of psychological management of tinnitus, which recommends that tinnitus intrusiveness, acceptance, mood, negative thoughts and beliefs, and sense of control be measured at a minimum. This approach would enhance the future possibility of a meta-analysis of this evidence.

### 4.3. Limitations

Limitations of this review include the exclusion of studies published in non-English languages, the conduct of the literature search by one author only, and the need to exclude several articles which were not obtained in full text. Additionally, no protocol was registered ahead of the review process, which is an important step in increasing transparency and reducing the potential risk of bias [77].

## 5. Conclusions

This scoping review mapped the current body of literature on Audiologist-provided CBT. The volume and range of the current evidence available were reported, as well as key characteristics of Audiologist-provided CBT programs described in the literature, and details relating to how research on this topic has been undertaken. This has led to the development of recommendations for future research. Currently, establishing the efficacy of Audiologist-provided CBT in comparison to Psychologist-provided CBT is considered a priority research question.

## Figures and Tables

**Table 1:** Characteristics of included sources.

Authors (Year)	Source Type	Aims as They Related to This Review	Study Design	Country of Conduct
Aazh et al. (2016) [29]	Research article	To assess patients’ opinions on the effectiveness of an Audiologist-provided CBT program for tinnitus and hyperacusis.	Service evaluation using cross-sectional surveys.	UK
Aazh and Moore (2018) [30]	Research article	To evaluate the efficacy of an Audiologist-provided CBT program for tinnitus and hyperacusis.	Service evaluation using a single group pre-test/post-test design.	UK
Aazh, Landgrebe, et al. (2019) [31]	Literature review	To review the body of literature relating to the efficacy of CBT for managing tinnitus and hyperacusis.	---	---
Aazh, Bryant, and Moore (2019) [32]	Research article	To assess participants’ views on the effectiveness and acceptability of an Audiologist-provided CBT program for tinnitus and hyperacusis.	Service evaluation using cross-sectional surveys.	UK
Beukes et al. (2015) [33]	Clinical trial protocol	Protocol for an RCT to assess the efficacy of Audiologist-guided iCBT in improving tinnitus outcomes.	---	---
Beukes et al. (2017) [34]	Research article	To assess the feasibility of an Audiologist-guided iCBT intervention for tinnitus prior to undertaking an RCT.	Single group pre-test/post-test design.	UK
Beukes, Baguley, et al. (2018) [35]	Research article	To assess the efficacy of an Audiologist-guided iCBT intervention for tinnitus vs. weekly monitoring.	Two-arm delayed intervention RCT.	UK
Beukes, Allen, et al. (2018) [36]	Research article	To investigate the long-term effects of an Audiologist-guided iCBT intervention for tinnitus at 1-year post intervention.	Repeated measures design.	UK
Beukes, Manchaiah, Baguley, et al. (2018) [37]	Research article	To identify processes which facilitate or hinder implementation of Audiologist-guided iCBT intervention for tinnitus.	Process evaluation ran parallel to an RCT (Beukes, Baguley, et al., 2018).	UK
Beukes, Manchaiah, Davies, et al. (2018) [38]	Research article	To explore participants’ experiences of an Audiologist-guided iCBT intervention for tinnitus.	Qualitative study using semi-structured telephone interviews.	UK
Beukes, Andersson, et al. (2018) [39]	Research article	To assess the efficacy of an Audiologist-guided iCBT intervention vs. Audiologist-provided individualised face-to-face care for tinnitus.	Two-arm non-inferiority RCT.	UK
Beukes et al. (2021a) [40]	Research article	To assess the feasibility of an Audiologist-guided iCBT intervention for tinnitus prior to undertaking an RCT.	Single group pre-test/post-test design.	USA
Beukes et al. (2021b) [41]	Research article	To determine the individual contribution of applied relaxation within an Audiologist-provided iCBT program for tinnitus.	Two-arm parallel RCT.	USA
Beukes, Andersson, Manchaiah, and Kaldo (2021) [42]	Book	To describe the use of CBT in tinnitus management and its application by Audiologists, and to disseminate CBT materials which can be used by audiologists.	---	---
Beukes, Andersson, and Manchaiah (2021) [43]	Research article	To identify processes which facilitate or hinder implementation of Audiologist-guided iCBT.	Process evaluation ran parallel to an RCT (Beukes, Andersson, Fagelson, and Manchaiah, 2022).	USA
Beukes, Andersson, Fagelson, and Manchaiah (2022) [44]	Research article	To assess the efficacy of an Audiologist-guided iCBT intervention for tinnitus vs. weekly monitoring.	Two-arm delayed intervention RCT.	USA
Beukes, Andersson, and Manchaiah (2022) [45]	Research article	To investigate the long-term effects of an Audiologist-guided iCBT intervention for tinnitus at 1-year post intervention.	Repeated measures design.	USA
Beukes and Manchaiah (Unpublished work) [46]	Book chapter	To provide an overview of CBT management of tinnitus and the potential role for Audiologists in guiding iCBT interventions.	---	---
Henry et al. (2009) [47]	Descriptive article	To introduce and describe ‘progressive audiological tinnitus management’, a multilevel intervention which can include Audiologist-provided CBT.	---	---
Henry et al. (2022) [48]	Text/opinion piece	To present the evidence for and against Audiologist-provided CBT for tinnitus and to make recommendations for practice.	---	---
McFerran and Baguley (2009) [49]	Text/opinion piece	To highlight arguments against, and barriers to, Audiologist-provided CBT.	---	---
Sweetow (1986) [50]	Descriptive article	To introduce and describe ‘tinnitus patient management’, an intervention which can include Audiologist-provided CBT.	---	USA
Sweetow (2000) [51]	Book chapter	To give an overview of CBT for tinnitus management, and to present arguments for Audiologist-provided CBT.	---	---
Tay (Unpublished work) [52]	Research article (under review)	To assess the efficacy of Audiologist-guided iCBT for tinnitus.	Non-inferiority single group pre-test/post-test design.	Indonesia
Taylor et al. (2017) [53]	Clinical trial protocol	Protocol for a psychologically informed, manualised, Audiologist-delivered intervention for tinnitus.	---	---
Taylor et al. (2020) [54]	Research article	To examine the acceptability and feasibility of an RCT of manualised Audiologist-provided psychological intervention (which includes CBT components).	RCT and post-test surveys and interviews to evaluate feasibility and acceptability.	UK
Thompson (2017) [55]	Text/opinion piece	To describe CBT as a management approach to tinnitus and to discuss the potential role of Audiologists in providing CBT.	---	---
Thompson et al. (2018) [56]	Research article	To determine which psychological components, including CBT techniques, Audiologists could and should provide.	A three-round Delphi survey.	UK
Tyler et al. (1989) [57]	Text/opinion piece	To advise on the assessment and management of tinnitus by Audiologists, including the potential role of Audiologists in providing CBT interventions.	---	---
Clinical trial registration ID CTRI/2020/10/028701 [58]	Clinical trial registration	To assess the acceptability and feasibility of an Audiologist-guided iCBT intervention for tinnitus after translation into three languages; Tamil, Kannada, and Hindi.	Proposed single group pre-test/post-test design.	India

Note. Abbreviations and acronyms used: CBT = cognitive behavioural therapy; iCBT = internet-based cognitive behavioural therapy; NHS = National Health Service; RCT = randomised controlled trial; UK = United Kingdom; USA = United States of America; vs. = versus.

## Appendix A

General Search String

1. Tinnitus [MeSH term]
2. Tinnit*
3. 1 OR 2
4. Audiologist-led
5. Audiologist-supported
6. Audiologist-delivered
7. Audiologist-guided
8. 4 OR 5 OR 6 OR 7
9. Cognitive behavioral therapy [MeSH term]
10. 1Cognit* AND behav*
11. 9 OR 10
12. 3 AND 8 AND 11

## Appendix B

Search String Used in PubMed

(“tinnitus”[MeSH Terms] OR “tinnit*”[Title/Abstract]) AND (“Audiologist-led”[Title/Abstract] OR “Audiologist-supported”[Title/Abstract] OR “Audiologist-delivered”[Title/Abstract] OR “Audiologist-guided”[Title/Abstract]) AND ((“cognit*”[Title/Abstract] AND “behav*”[Title/Abstract]) OR “cognitive behavioral therapy”[MeSH Terms])

## Appendix C

Data Extraction Form

Title of paper:	
Authors:	
Year of publication:	
Country of Origin:	
Type of source:	
Aims/purpose:	
Population and sample size within the source of evidence (if applicable): - Age - Gender - Tinnitus details	
Methodology/methods:	
Intervention type, comparator, and details of these: - Who delivered? - Training - Setting - Mode of delivery - Duration/frequency - Content - Comparators	
Outcomes measured, when they were measured, and how they were measured (if applicable):	
